# Supplementary material for: Molecular evidence and ecological niche modeling reveal an extensive hybrid zone among three Bursera species (section Bullockia)
Source: PLoS One. 2021 Nov 19;16(11):e0260382. doi: 10.1371/journal.pone.0260382 (PMC8604287; doi:10.1371/journal.pone.0260382)

Molecular evidence and ecological niche modeling reveal an extensive hybrid zone among three *Bursera* species (Section *Bullockia*)

Eduardo Quintero Melecio, Yessica Rico, Andrés Lira Noriega, Antonio González Rodríguez

S1 Figure. BEAST phylogenetic tree of the concatenated ETS and PEPC nuclear genes

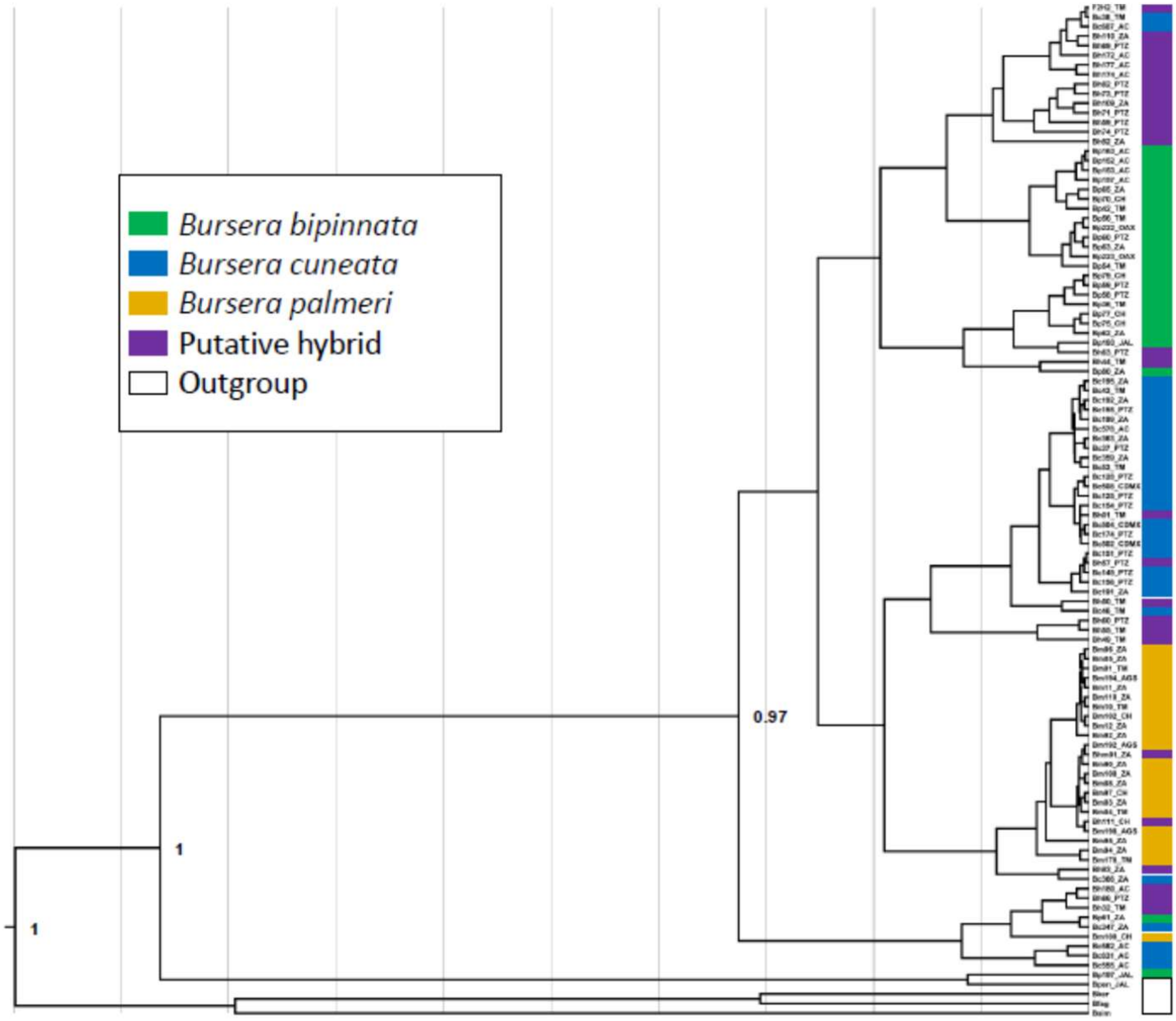

Supplement: S1 Fig — The tree shows the relationships between the tree Bursera species and the putative hybrids. Outgroup species were B. simaruba, B. kerberi, and B. fagaroides. Values above nodes represent posterior probabilities. (PDF) [file pone.0260382.s001.pdf]
