# Supplementary material for: Molecular evidence and ecological niche modeling reveal an extensive hybrid zone among three Bursera species (section Bullockia)
Source: PLoS One. 2021 Nov 19;16(11):e0260382. doi: 10.1371/journal.pone.0260382 (PMC8604287; doi:10.1371/journal.pone.0260382)

Molecular evidence and ecological niche modeling reveal an extensive hybrid zone among three *Bursera* species (Section *Bullockia*)

Eduardo Quintero Melecio, Yessica Rico, Andrés Lira Noriega, Antonio Gonzále-Rodríguez

S2 Fig. Predicted suitable areas for each *Bursera* species through ecological niche modeling.

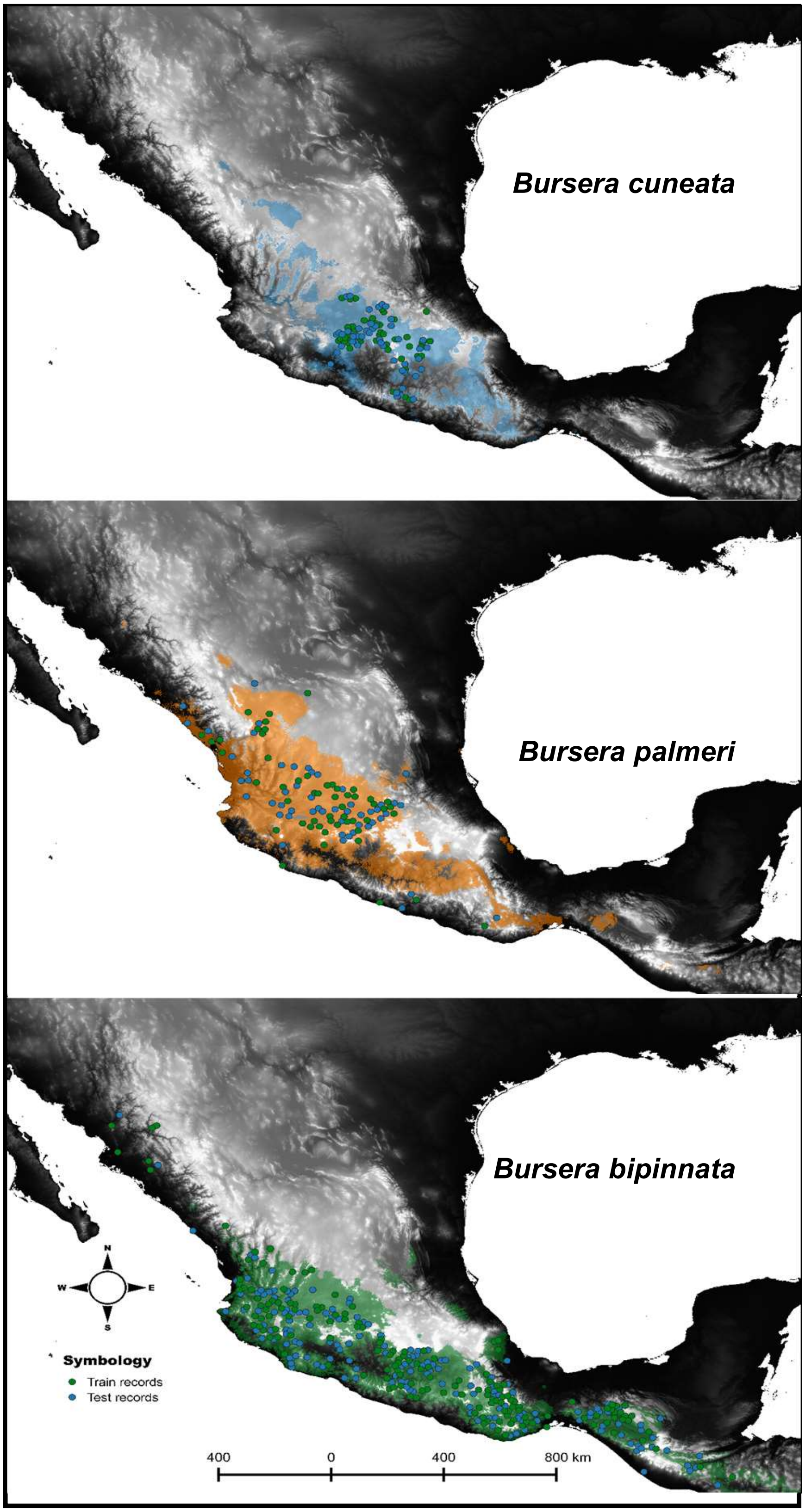

Supplement: S2 Fig — The model was constructed based on the best calibration based on Kuenm. Green dots represent the training set and blue dots the testing set. The black and gray shading represent an altitudinal gradient with lighter areas being altitudinally higher. (PDF) [file pone.0260382.s002.pdf]
