# Supplementary material for: Molecular evidence and ecological niche modeling reveal an extensive hybrid zone among three Bursera species (section Bullockia)
Source: PLoS One. 2021 Nov 19;16(11):e0260382. doi: 10.1371/journal.pone.0260382 (PMC8604287; doi:10.1371/journal.pone.0260382)

Molecular evidence and ecological niche modeling reveal an extensive hybrid zone among three *Bursera* species (Section *Bullockia*)

Eduardo Quintero Melecio, Yessica Rico, Andrés Lira Noriega, Antonio Gonzále-Rodríguez

S3 Fig. Results from the similarity test between the putative hybrid and each of the parental *Bursera* species.

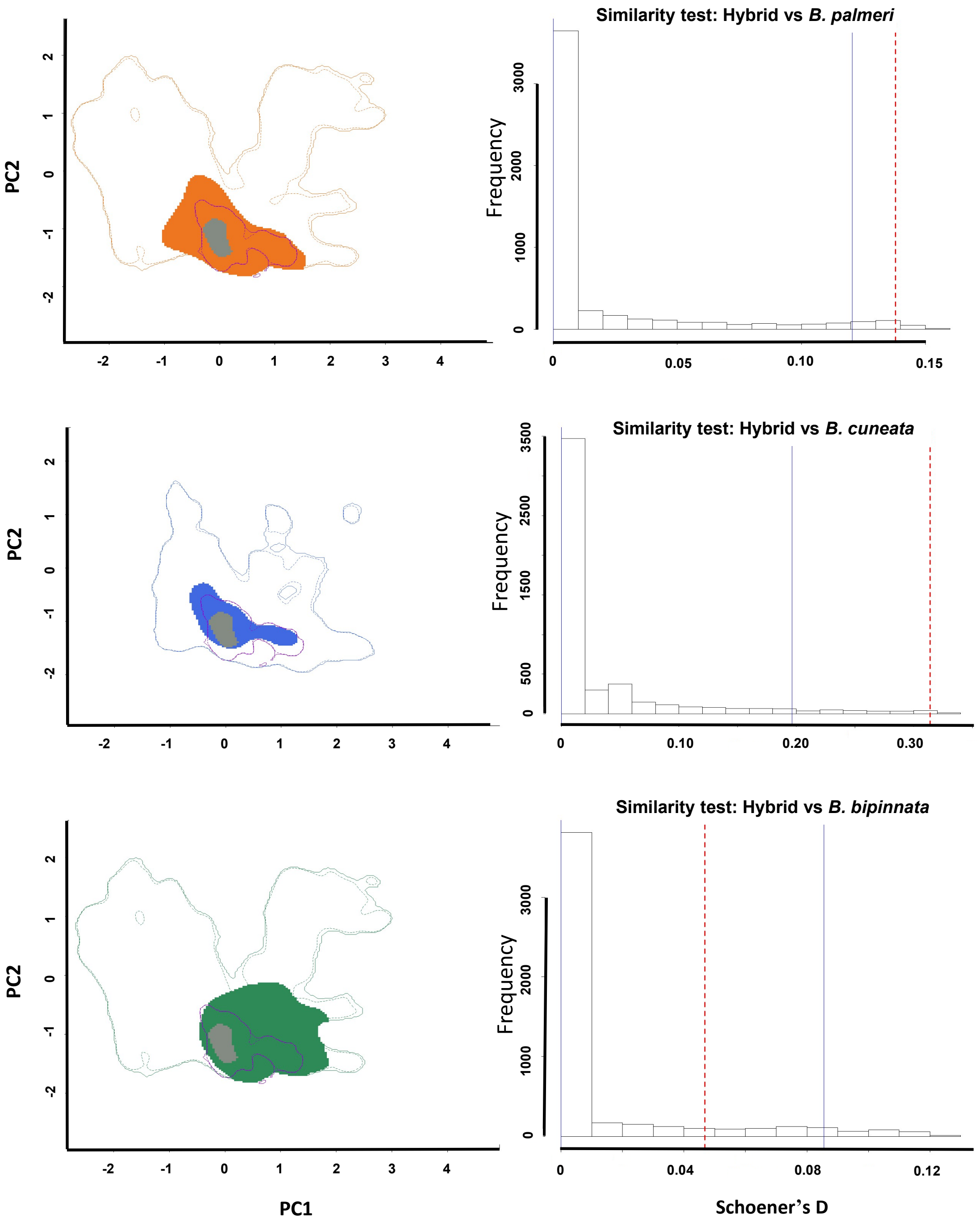

Supplement: S3 Fig — The left panel shows the environmental space of Bursera palmeri (orange), B. cuneata (blue) and B. bipinnata (green) and the putative hybrids (purple). The gray area denotes the overlap area. The right panel shows the distribution of observed vs expected values of Schoener’s D, the blue lines denote the range of 95% of expected values and the red lines the observed values. (PDF) [file pone.0260382.s003.pdf]
