# Supplementary material for: Molecular evidence and ecological niche modeling reveal an extensive hybrid zone among three Bursera species (section Bullockia)
Source: PLoS One. 2021 Nov 19;16(11):e0260382. doi: 10.1371/journal.pone.0260382 (PMC8604287; doi:10.1371/journal.pone.0260382)

Molecular evidence and ecological niche modeling reveal an extensive hybrid zone among three *Bursera* species (Section *Bullockia*)

Eduardo Quintero Melecio, Yessica Rico, Andrés Lira Noriega, Antonio Gonzále-Rodríguez

S4 Fig. Density plots for the CCSM4 models showing the altitudinal range values predicted for the overlap niche zone (i.e., hybrid zone) for each climatic niche scenario.

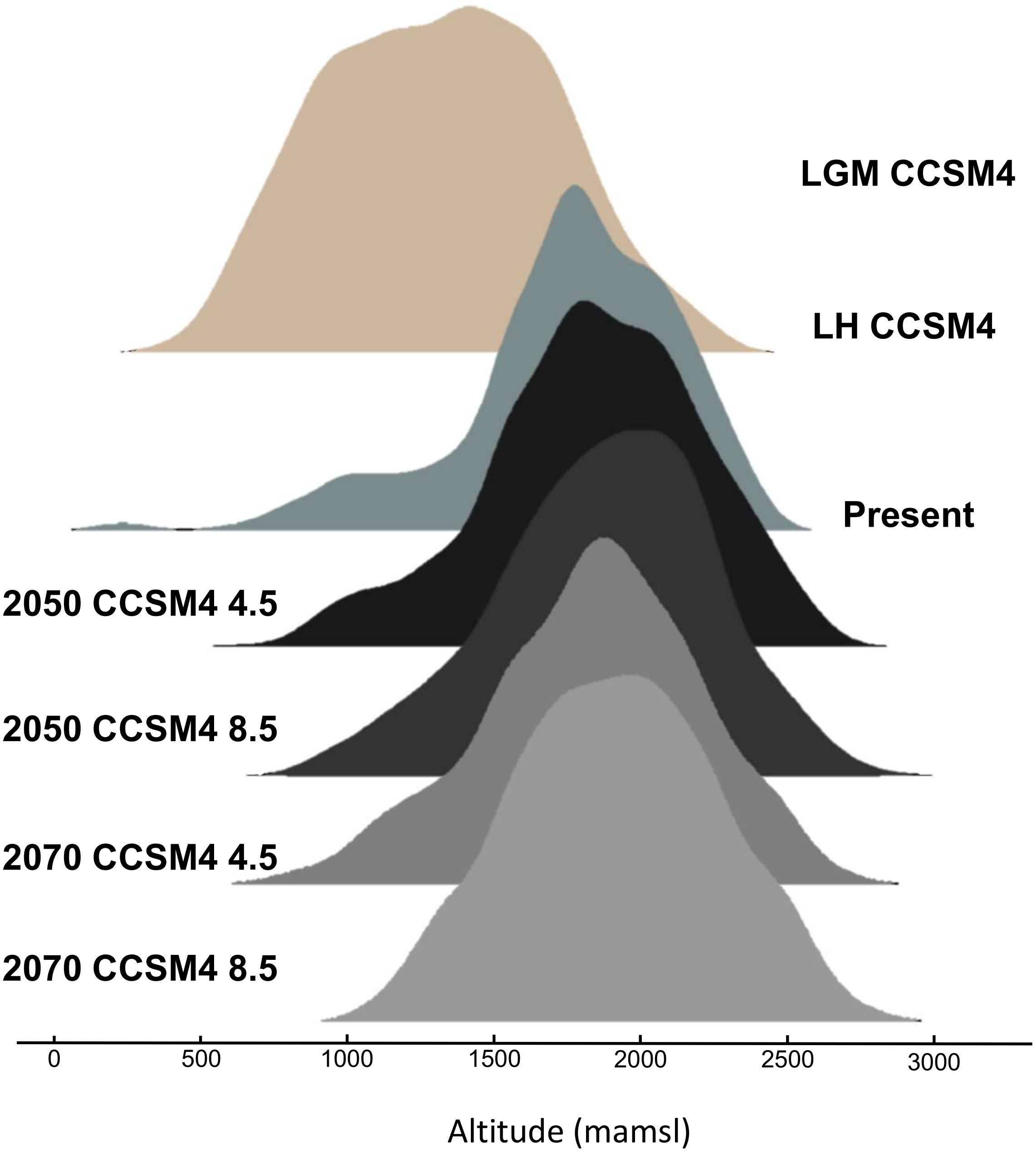

Supplement: S4 Fig — Scenarios: LGM Last Glacial Maximu, LH Late Holocene. Values of the x-axis denotes the elevational ranges. (PDF) [file pone.0260382.s004.pdf]
