# Supplementary material for: Molecular evidence and ecological niche modeling reveal an extensive hybrid zone among three Bursera species (section Bullockia)
Source: PLoS One. 2021 Nov 19;16(11):e0260382. doi: 10.1371/journal.pone.0260382 (PMC8604287; doi:10.1371/journal.pone.0260382)
Supplement: S2 Table — All comparisons were statistically significant P = 0.0001. (PDF) [file pone.0260382.s007.pdf]

Molecular evidence and ecological niche modeling reveal an extensive hybrid zone among three *Bursera* species (Section *Bullockia*)

Eduardo QuinteroMelecio, Yessica Rico, Andrés Lira Noriega, Antonio González Rodríguez

S2 Table. Pairwise F_{ST} (below) and G_{ST} (above) genetic distances among the three *Bursera* species and the putative hybrids. All comparisons were statistically significant *P* = 0.0001

	B. cuneata	***B. palmeri***	***B. bipinnata***	***Putative hybrid***
B. cuneata	-	0.275	0.08	0.062
B. palmeri	0.526	-	0.219	0.16
B. bipinnata	0.304	0.545	-	0.019
**Putative hybrid**	0.135	0.503	0.218	-
