# Supplementary material for: Molecular evidence and ecological niche modeling reveal an extensive hybrid zone among three Bursera species (section Bullockia)
Source: PLoS One. 2021 Nov 19;16(11):e0260382. doi: 10.1371/journal.pone.0260382 (PMC8604287; doi:10.1371/journal.pone.0260382)
Supplement: Attachment — Submitted filename: Response letter.pdf [file pone.0260382.s010.pdf]

Response letter

Thank you for submitting your manuscript to PLOS ONE. After further consideration, we feel that it has been vastly improved after revision, but does not fully meet PLOS ONE's publication criteria as it currently stands. Therefore, we invite you to submit a revised version of the manuscript.

The authors have done a solid job is responding to the reviewers' comments and criticisms. However, as I read through the revised MS, I found a large number of grammatical errors, misuses of commas and parentheses, and very long sentences that needed to be broken up. Since one of the requirements for publication in this journal is that all manuscripts be presented in proper English, I have returned the MS to the authors with edits so they can improve the presentation of their work for their readers. The paper is still quite long for the message, but I leave it to the authors to attempt to delete unneeded sentences and duplicate information.

R= We are very thankful with the comments made by the academic Editor, William J. Etges, who made considerably edits of grammatical errors and other style issues. We have made all the suggested changes including broken up some large sentences. Moreover, we have considerably reduced the number of sentences, mostly for the discussion section where the manuscript was too long. The sentences included unnecessary information or duplicated information with introduction and discussion. A detailed record of the changes made can be revised in the manuscript with track changes.
